# Outcomes and Prognostic Factors of Salvage Radiation for Postoperative Lymph Node Recurrence of Esophageal Squamous Cell Carcinoma

**DOI:** 10.3389/fonc.2021.638521

**Published:** 2021-03-19

**Authors:** Chi Zhang, Xiao-Lin Ge, Chen-Jun Huang, Shu Zhang, Xin-Chen Sun

**Affiliations:** ^1^Department of Radiation Oncology, The First Affiliated Hospital With Nanjing Medical University, Nanjing, China; ^2^Department of Thoracic Surgery, The First Affiliated Hospital With Nanjing Medical University, Nanjing, China

**Keywords:** esophageal squamous cell carcinoma, lymph node recurrence, salvage radiotherapy, chemoradiotherapy, prognostic factors

## Abstract

**Purpose:** Recurrence of esophageal squamous cell carcinoma (ESCC) in regional lymph nodes (LNs) after surgical section can be treated with salvage resection, radiotherapy (RT) or chemoradiotherapy (CRT). RT or CRT is more widely used in clinic. This paper investigates the effects, toxicities and prognostic risk factors of salvage RT or CRT on patients with LN recurrence.

**Methods:** We retrospectively analyzed the clinical outcomes of 103 patients receiving salvage RT or CRT for LN recurrence after ESCC resection. In total, 39 patients received RT alone and 64 received concurrent CRT. All the patients received intensity modulated radiation therapy (IMRT), administered with a median dose of 62 Gy (range, 50–70 Gy).

**Results:** The median follow-up time was 44.5 months, and median survival was 22.5 months (5.5–99.5 months). One-, 3-, and 5-year overall survival (OS) were 80.6, 37.0, and 25.8%, respectively. One- and 2-year progression free survival (PFS) were 57.3 and 34.0%, respectively. Grade 3 or above toxicity was low (16.5%) and no treatment-related deaths occurred. In univariate analysis of OS, pN0 (*p* = 0.039), smaller LN volume (≤25 cm^3^, *p* = 0.019), combined chemotherapy (*p* = 0.041) and single LN recurrence (*p* = 0.001) were associated with prolonged OS. And pT1-2 (*p* = 0.044), pN0 (*p* = 0.042), irradiation dose (>60 Gy, *p* = 0.044), combined chemotherapy (*p* = 0.019) and single LN recurrence (*p* = 0.002) were associated with prolonged PFS. In multivariate analysis, the patients with only one recurrent node had a significant better OS (HR = 0.556, 95% CI 0.324–0.956, *p* = 0.034) and PFS (HR = 0.528, 95% CI 0.339–0.847, *p* = 0.008).

**Conclusions:** Salvage RT or CRT for regional LN recurrence is effective and acceptable. Fewer recurrent nodes may indicate a better long-term survival.

## Introduction

Esophagectomy is the primary treatment choice for resectable esophageal squamous cell carcinoma (ESCC) (1). However, nearly half patients may experience recurrence, which adversely affects survival (2–4). ESCC recurs frequently in lymph nodes (LNs) within 1–2-year after radical surgery, and its 5-year survival rate is still low (15 to 39%). The most common local recurrence sites are anastomotic, supraclavicular lymph nodes and mediastinal lymph nodes. Isolated LN recurrence brings a longer survival than local relapse or distant metastasis (5–7). Chemoradiotherapy (CRT) is considered to be an effective and feasible modality if ESCC recurs outside of previously radiated fields. Patients who are intolerant to CRT can switch to radiotherapy (RT) (8–10). Salvage resection can be an option in selected patients by removing recurrent supraclavicular lymph nodes. Given that chemotherapy might also enhance the radiosensitivity, several studies have demonstrated that concurrent CRT could promote locoregional control and reduce distant recurrence. However, the prevailing data on this treatment is not conclusive, and controversy remains regarding the therapeutic strategies. Herein, we retrospectively analyzed the clinical outcomes of 103 patients who had undergone esophagectomy and later developed LN recurrence to identify the preferable treatment options and predictive factors.

## Materials and Methods

### Study Population

The retrospective study included consecutive patients undergoing salvage radiotherapy for postoperative LN recurrence between August 2011 and June 2017 at the People's hospital of Jiangsu Province, China. Inclusion criteria: (1) pathologically confirmed ESCC; (2) R0 resection with two-incision or three-incision esophagectomy; (3) only LN recurrence without other recurrence patterns or distance metastasis; (4) Kanofsky Performance Status (KPS) ≥ 70; (5) LNs recurrence was diagnosed by two experienced physicians in pathology or radiology, and the imaging evaluation should include the supraclavicular, mediastinal and gastric lymphatic drainage area. Some cases with palpable supraclavicular lymph node recurrences were pathologically confirmed. Suspicious nodes without pathological diagnosis were diagnosed by two consecutive CT, which showed the suspicious nodes continued to increase or the recurrent nodes with a short diameter >1 cm. In some cases, 18F-PET/CT or chest MR enhancement scanning was recommended. The volume of recurrent nodes was measured by radiologists. The software used for tumor image segmentation is ITK-SNAP version 3.6. The demographics and tumor characteristics were displayed in details (Table 1). This study was approved by the Ethics Committee of Jiangsu Province People's Hospital and conducted in accordance with the Helsinki Declaration. Written informed consent was collected from all the patients.

**Table 1:** Baseline characteristics and univariate analyses for overall survival and progression free survival.

		**OS**		**PFS**	
	**No./percent**	**χ2**	***p-value***	**χ2**	***p-value***
**Gender**					
Female	22 (21.3%)	0.289	0.591	0.297	0.586
Male	81 (78.6%)				
**Age, year (44–82)**					
≤60	39 (37.9%)	1.017	0.313	0.854	0.355
>60	64 (62.1%)				
**Tumor location**					
Upper	29 (28.2%)	0.219	0.896	0.872	0.274
Middle	35 (33.9%)				
Lower	39 (37.9%)				
**pT stage**					
pT1/pT2	52 (50.5%)	1.204	0.282	4.039	0.044^*^
pT3/pT4	51 (49.5%)				
**pN stage**					
pN0	61 (59.2%)	4.266	0.039^*^	4.121	0.042^*^
pN+	42 (40.8%)				
**Differentiation**					
Well	1 (0.97%)	0.571	0.752	0.274	0.872
Moderate	58 (56.3%)				
Poor	44 (42.7%)				
**Adjuvant therapy**					
Without	62 (60.2%)	1.965	0.161	1.575	0.210
With	41 (39.8%)				
**Recurrence location**					
Supraclavicular	34 (33.0%)	2.655	0.448	4.228	0.238
Mediastinal	66 (64.1%)				
Abdominal	1 (0.97%)				
Mixed	2 (1.94%)				
**No. of LN metastasis**					
Mono	60 (58.3%)	11.69	0.001^*^	10.05	0.002^*^
Multiple	43 (41.7%)				
**max LN diameter**					
≤3 cm	52 (50.5%)	3.037	0.081	0.550	0.459
>3 cm	51 (49.5%)				
**Volume of LN**					
≤25 cc	55 (53.4%)	5.524	0.019^*^	3.615	0.057
>25 cc	48 (46.6%)				
**Irradiation Dose**					
≤60 Gy	40 (38.8%)	3.602	0.058	4.062	0.044^*^
>60 Gy	63 (61.2%)				
**Recurrence interval**					
≤16 months	49 (47.6%)	0.013	0.908	0.004	0.947
>16 months	54 (52.4%)				
**Chemotherapy**					
Without	39 (37.9%)	4.178	0.041^*^	5.491	0.019^*^
With	64 (62.1%)				

*LN, lymph node; OS, overall survival; PFS, progression free survival*.

* *p < 0.05*.

### Treatment

Target area delineation: Gross tumor volume (GTV) was defined as recurrent nodes, and the planning target volume (PTV) of GTV, namely P-GTV was defined as GTV plus a margin of 5 mm. Clinical target volume (CTV) included GTV with a 5 mm margin expansion and drainage regions of recurrent nodes. PTV was delineated from a CTV with a marginal expansion by 0.5 cm. All the patients received intensity-modulated radiotherapy (IMRT) with conventional fraction, and P-GTV: a median dose of 62 Gy (range, 50–70 Gy), PTV: 50 Gy. For the areas that have been irradiated, it must be at least 1-year interval for salvage radiotherapy. Concurrent chemotherapy regimens were composed of paclitaxel + platinum, raltitrexed + platinum or Tegafur Gimeracil Oteracil Potassium Capsule (S-1) alone. The first regimen was comprised of intravenous infusion of paclitaxel (135 mg/m^2^) on day 1 plus nedaplatin (70 mg/m^2^) on day 1 or cisplatin (75 mg/m^2^) averaged on days 1–3 for a 21-day cycle. The second regimen consisted of raltitrexed (3 mg/ m^2^) on day 1 plus nedaplatin (70 mg/m^2^) on day 1 or cisplatin (75 mg/m^2^) averaged on days 1–3 for a 21-day cycle. The third regimen was S-1 for oral administration on day 1–14 for a 21-day cycle.

### Follow-Up

One month after radiotherapy, barium meal examination and chest CT was conducted to assess tumor response according to Response Evaluation Criteria in Solid Tumors (RECIST 1.1). Patients were followed up at intervals of 3 to 6 months after that, including medical history, barium meal, ultrasonography, chest and abdomen CT and PET/CT, and the cause of death was recorded. Progression free survival (PFS) was defined as the interval from end of the radiotherapy to locoregional relapse or distant metastasis. Overall survival (OS) was defined as the interval from the end of radiotherapy to death or last follow-up. Local failure free survival (LFS) was defined as the interval from the end of the radiotherapy to locoregional relapse. Distant metastasis free survival (DMFS) was defined as the interval from the end of the radiotherapy to distance metastasis. Follow-up statistics were reviewed by September 30, 2019.

### Statistical Analysis

All statistical analyses were performed using the SPSS Statistics 22.0 (Chicago, IL, USA). Categorical variables in baseline characteristics were compared by the chi-square test or Fisher's exact test. OS were estimated with the Kaplan–Meier method, and the differences in survival in the univariate analysis were assessed with the log-rank test. Variables with significance of <0.1 on univariate analysis were applied to Cox's proportional hazards regression analysis. Two-tailed *P* < 0.05 was considered statistically significant.

## Results

### General Information

A total of 103 patients (81 males and 22 females) who underwent radical esophagectomy between August 2003 and May 2017 were enrolled in our study. Among them, 29 patients were treated with esophagectomy in the upper thoracic segment, 35 in the middle segment, and 39 in the lower segment. After surgery 23 patients had received adjuvant RT. Median interval from surgery to LN recurrence was 16 months (1–151 months). The recurrent nodes were mostly distributed in the supraclavicular and mediastinum regions. The median volume of these nodes was 22.07 cm^3^ (1.73–304.9 cm^3^).

All patients completed salvage radiation, and 64 (62.1%) of patients received concurrent chemotherapy: paclitaxel plus platinum (26 patients), combination of raltitrexed with platinum (26 patients), and S-1 (12 patients). Other characteristics were detailed in Table 1, which were included in the univariate analysis to evaluate their prognostic impact. The parameters were cut off according to the median values of age, recurrent lymph node size, and recurrence interval.

### Treatment Outcome

The median follow-up time was 44.5 months for 30 living patients, and median OS after salvage RT was 22.5 months (5.5–99.5 months). The overall tumor response rate was 73.8%. To be specific, 18 patients showed complete response (CR) and 67 patients showed partial response (PR). During the follow-up, there were 29 distant metastasis, 38 local progression and 6 multiple type of relapses after radiation. Sixty-four patients died from the tumor progression. Five patients died of other reasons and four died of unknown reasons. Detailed cause of death was shown in Supplementary Table 1. Patients with local failure mostly died of tumor invasion-caused hemorrhage or tumor compression-caused suffocation. The 1- and 2-year LFS was 62.1 and 35.9%, and 1- and 2-year DMFS was 61.2 and 37.5%. Thirty patients were alive at the last follow-up, but nine of these had a recurrence of cancer. One-, 3-, and 5-year OS were 80.6, 36.7, and 25.8%, respectively.

### Univariate and Multivariate Analyses of Prognostic Factors for Survival

**Figure 1:**
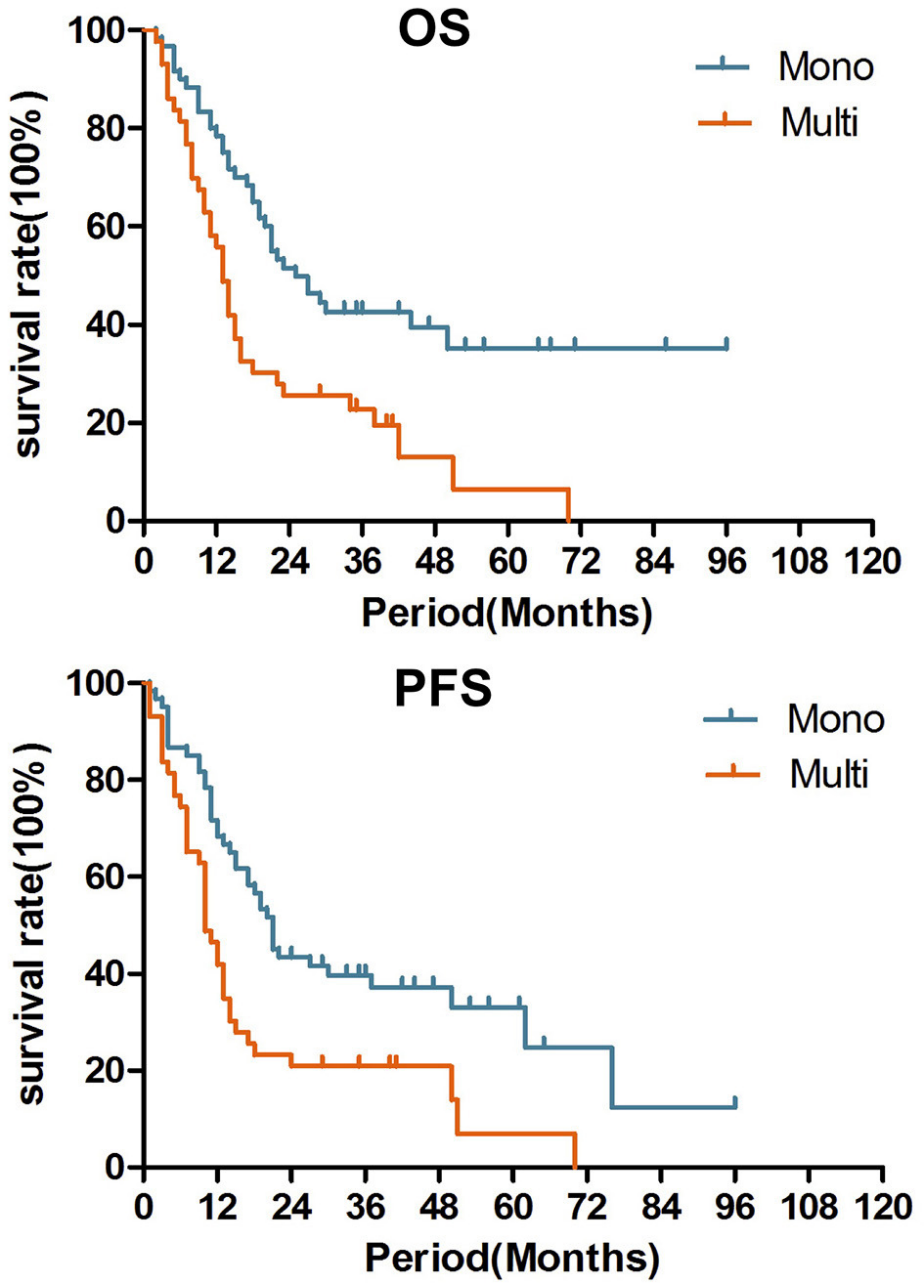
Over survival and Progression free survival after salvage radiotherapy for single recurrent lymph node and multiple lymph nodes (*p* = 0.001, 0.008).

In the univariate analysis of OS, pN0 (*p* = 0.039), smaller LN volume (≤25 cm^3^, *p* = 0.019), combined chemotherapy (*p* = 0.041) and lower LN recurrence (one node, *p* = 0.001) were associated with prolonged OS. In the univariate analysis of PFS, pT1-2 (0.044), pN0 (*p* = 0.042), irradiation dose (>60 Gy, *p* = 0.044), combined chemotherapy (*p* = 0.019) and lower LN recurrence (one node, *p* = 0.002) were associated with prolonged PFS (shown in Table 1). In the multivariate analysis incorporating pT stage, pN stage, LN volume, LN number, radiation dose and CRT (*p* < 0.1), only recurrent LN number (single vs. multiple, HR = 0.556, 95% CI 0.324–0.956, *p* = 0.034) was a significant prognostic factor (Table 2; Figure 1). The median OS were 28.5 months with single LN recurrence and 17.0 months with multiple LN recurrence. The median PFS were 21.5 months with single LN recurrence and 10.0 months with multiple LN recurrence.

**Table 2:** Multivariate analyses for OS and PFS.

		**OS**		**PFS**	
	**No**.	***HR (95% CI)***	***p***	***HR (95% CI)***	***p-value***
**pT stage**					
pT1/pT2	52	1.204	0.282	0.716 (0.446–1.148)	0.166
pT3/pT4	51				
**pN stage**					
pN0	61	0.669 (0.407–1.225)	0.113	0.716 (0.441–1.162)	0.176
pN+	42				
**No. of LN metastasis**					
Mono	60	0.556 (0.324–0.956)	0.034^*^	0.528 (0.339–0.847)	0.008^*^
Multiple	43				
**Irradiation Dose**					
≤60 Gy	40	1.438 (0.885–2.335)	0.142	1.561 (0.706–3.452)	0.272
>60 Gy	63				
**Chemotherapy**					
Without	39	1.240 (0.760–2.025)	0.389	1.357 (0.835–2.204)	0.218
With	64				

*LN, lymph node; OS, overall survival; PFS, progression free survival*.

* *p < 0.05*.

### Adverse Events

The major acute events were radiation esophagitis and neutropenia, mostly classified as Grade 1 or 2 toxicities. Hematologic and non-hematologic adverse events were recorded, according to the Common Terminology Criteria for Adverse Events (CTCAE) v4.0 (shown in Table 3). Grade 3 or 4 neutropenia was observed in 7 patients (7/64) in CRT group and two (2/39) in RT group. Grade 1 or 2 radiation pneumonitis was observed during or shortly after the treatment. Only two patients need to treat with steroid and antibiotics. Anastomotic stricture was the most common late-stage event, found in four patients (3.9%). There was no unexpected augment in serious adverse events or therapy-related deaths.

**Table 3:** Acute or late G3/4 Toxicity.

**Toxicity**	**G3**	**G4**
**Acute toxicity**	**Num. (percentage)**	**Num. (percentage)**
Neutropenia	7 (6.80%)	2 (1.94%)
Thrombocytopenia	2 (1.94%)	0
Anemia	0	0
Esophagitis	2 (1.94%)	0
Radiation pneumonia	0	0
**Late toxicity**		
Anastomotic stricture	4 (3.88%)	
Heart	0	0

*This percentage are determined by the number of cases over the total patients*.

## Discussion

According to the previous studies of salvage radiation for recurrent LN after ESCC resection, the median survival and a 3-year OS has been greatly improved in the past 5-years (5, 8, 11–13). The studies in the 2000s showed the median survival was only 10.5 months (range, 7–20.3 months) (14–20), while that in the past 5 years has increased to 22.0 months (range, 12–29.9 months) (shown in Table 4). In our study, radiotherapy achieved favorable results, as evidenced by the OS and median survival. The 3-year survival rate was 36.7%, and median OS was 22.5 months, respectively. Grade 3 or above toxicity was low (16.5%) and no treatment-related deaths occurred. In the multivariate analysis, solitary recurrent node showed potential as an independent prognostic factor (*p* = 0.034). Our results suggested that radiotherapy, supported with or without chemotherapy, is an effective and feasible salvage treatment for LN recurrence after radical resection of ESCC.

**Table 4:** Previous studies on salvage RT with lymph node recurrence.

**Author**	**Num**	**Regimen**	**MST (month)**	**1-y OS**	**2-y OS**	**3-y OS**	**5-y OS**
Jingu (5)	30	60 Gy + FP	39	60.6%		56.3%	
Shioyama (12)	82	45–70.5 Gy + FP/5-FU	7		22%		11%
Nakamura (8)	22	46–66 Gy + FP	20.3			26.6%	
Lu (13)	42 !!break 31	60 Gy alone !!break 60 Gy + FP	9 17	33.8% !!break 62.5%		0 !!break10.5%	
Zhang (20)	50	50.4–64 Gy + FP/TP	13.3	56%		14%	
Zhang (14)	27	50–60 Gy + FP	26	88.9%	60.2%		
Jingu (17)	80	50–70 Gy + FP/TP	26.5			39.8%	30.5%
Kimoto (16)	35	60 Gy +P/S-1	29.9		55.7%		
Yamashita (15)	237	45–70 Gy + FP/TP	21.6			37%	
Kawamoto (18)	57	60–66 Gy + FP/DOC	22		43.7%	36.9%	27.6%
Chen (19)	83	50–66 Gy + TP/PF	18	83.0%	57.1%	40.1%	35.1%

*Num, number; LN, lymph node recurrence; LR, local recurrence; FP, Fluorouracil+platinum; TP, Paclitaxel+platinum; 5-FU, 5-Fluorouracil; DOC, Docetaxel*.

Retrospective studies reported that survival increased with radiation dose [(11, 16). Zhang et al. (20) showed that doses higher than 60 Gy improved PFS and OS (median OS of 16.3 months, *p* = 0.041) Ma et al. (6) reported an encouraging result of median OS of 35 months in CRT group and that of 19 months in RT group at a radiation dose of 62–70 Gy. Kawamoto et al. (18) reported a median OS of 22 months after using a radiation dose of 60–66 Gy. Fortunately, new radiotherapy technology has emerged with reduced serious adverse events. IMRT was commonly used in our study. According to our results, patients who received dose higher than 60 Gy tended to have a longer survival (median OS of 26.5 months) than those who received a lower dose (median OS of 16.5 months), however, no significant difference was observed after multivariate analysis (*p* = 0.142). All patients with severe toxicity (Grade 3 anastomotic stenosis and grade 4 gastric-bronchial fistula) received a higher dose (P-GTV: 64–66 Gy). Liu et al. (21) reported that the percent of the gastric tube volume receiving at least 50 Gy (V_50_) was strongly associated with the degree of toxicity. Recurrent lymph nodes probably need a higher dose, because they may be more radioresistant than primary tumor. Although no consensus has been reached about the optimal radiation dose, a high dose above 60 Gy may be suitable for those without radiation history and recurrent nodes that were not close to thoracostomach.

Jingu et al. (17) showed by matched-pair analysis that elective nodal irradiation could not improve, or even worsen OS and irradiation-field control rate in chemoradiotherapy for postoperative loco-regional recurrent esophageal cancer (15, 22). Similar findings suggested that no difference in survival between elective node irradiation and involved field irradiation in patients with lymph node oligo-recurrence, but bring with acute adverse events related to higher toxicity (Grade 3 or 4).

There were three patients who underwent out-field relapse received a second salvage radiation. Given that tumor is more radioresistant in patients with a radiation history, we should probably use a higher re-radiation dose. However, less biological effective dose (BED) was used in these patients than those without a radiation history, due to the potential toxicity of re-radiation. Jingu et al. (23) also reported that re-radiation for formerly radiated recurrent lymph nodes might be applicable but unsatisfactory, with a median survival of 16.0 months and a 3-year OS of 17.9%. IMRT was commonly used in our study, and in IFI therapy, a higher BED could be given and safety be guaranteed simultaneously in patients who received re-radiation, avoiding severe toxicity. The esophageal and thoracostomach hemorrhage both happened in re-radiation group, but these were assumed by tumor invasion. So, the toxicity of re-radiation in our study was acceptable.

Concurrent radio-chemotherapy is thought to improve loco-regional control and survival by enhancing the tumor response rate and reducing the metastasis. Lu et al. (13) suggested that CRT could be well-tolerated and improved the survival synergistically, for the OS at 1- and 3-years was 62.5 and 10.5% in the CRT group and 33.8 and 0% in the RT group. Yamashita et al. (15) reported the 3-year OS was 39.7% in CRT group and 20.8% in RT group (*p* < 0.05) in 237 patients. Zhang et al. (20) and Bao et al. (24) reported much better survival realized by RT combined with a taxane-based regimen than RT combined with FP. In our study, the 3-year OS was 41.8%, and the median OS was 24.5 months in CRT group, while these parameters became 24.6% and 16.5 months in RT group (*p* = 0.041). However, after multi-analysis, we did not find the efficacy of concurrent chemotherapy. It could be due to a high rate of local failure, and we retrospectively found some patients receiving CRT who died of local failure in the short term, especially tumor-caused hemorrhage and suffocation. But during the follow-up, we observed a longer median interval to distant metastasis in the CRT group. For those patients with disseminated micro-metastasis around the LNs, local and systemic therapy may be still needed to improve the overall control. Chen et al. (19) reported that concurrent CRT had a better response rate (91.4%) than RT alone (73.2%), but a worse median survival (16 vs. 22 months, *p* = 0.570). But his study was not randomized. Given the bias in subject selection, the tumor load in concurrent CRT group was significantly greater than the RT group. As a retrospective analysis with a small sample size, its reliability should be warranted with larger-sample prospective studies.

## Conclusion

Salvage radiotherapy with or without chemotherapy for postoperative LN recurrence is safe and effective. Fewer recurrent nodes indicate longer OS. The optimal treatment strategy (irradiation dose and chemotherapy regimen) should be warranted with studies of larger sample sizes.

## Data Availability Statement

The raw data supporting the conclusions of this article will be made available by the authors, without undue reservation.

## Ethics Statement

The studies involving human participants were reviewed and approved by Ethics Committee of Jiangsu Province People's Hospital. The patients/participants provided their written informed consent to participate in this study.

## Author Contributions

CZ is responsible for data analysis and article writing. X-LG is responsible for data collection and article writing. C-JH is responsible for data collection and patient follow-up. SZ is responsible for experiment design and article review. X-CS is responsible for experiment design. All authors contributed to the article and approved the submitted version.

## Conflict of Interest

The authors declare that the research was conducted in the absence of any commercial or financial relationships that could be construed as a potential conflict of interest.
