# Supplementary material for: Outcomes and Prognostic Factors of Salvage Radiation for Postoperative Lymph Node Recurrence of Esophageal Squamous Cell Carcinoma
Source: Front Oncol. 2021 Mar 19;11:638521. doi: 10.3389/fonc.2021.638521 (PMC8017315; doi:10.3389/fonc.2021.638521)
Supplement: Supplementary file 1 [file Table_1.doc]

**Supplementary Table 1. The cause of death**

| Cause of death | Number (percentage) |
| --- | --- |
| Local progression | 34 (46.6%) |
| Death of Suffocation | 11 |
| Death of Hemorrhage | 14 |
| Death of Leakage | 5 |
| Death of dysphagia | 4 |
| Distant metastasis | 24 (32.8%) |
| Death of Lung | 10 |
| Death of Liver | 6 |
| Death of Multi-site | 8 |
| Death of Multiple failure | 6 (8.2%) |
| Death of Other diseases | 5 (6.8%) |
| Death of Unknown reason | 4 (5.5%) |

**This percentage determined by the number of cases over the number of all the dead patients.**
